# Palliative radiotherapy combined with stent insertion to relieve dysphagia in advanced esophageal carcinoma patients: A systematic review and meta−analysis

**DOI:** 10.3389/fonc.2022.986828

**Published:** 2022-09-12

**Authors:** Zhiyuan Xu, Haonan Liu, Shengli Li, Zhengxiang Han, Jingjing Chen, Xiangting Liu, Qiang Li, Hong Mu, Jiaqi Yuan, Hailong Lu, Peisheng Jin, Xianliang Yan

**Affiliations:** ^1^ Xuzhou Medical University, Xuzhou, Jiangsu, China; ^2^ Department of Emergency, Affiliated Hospital of Xuzhou Medical University, Xuzhou, China; ^3^ Department of Oncology, Affiliated Hospital of Xuzhou Medical University, Xuzhou, China; ^4^ Department of Medical Record Statistics, Affiliated Hospital of Xuzhou Medical University, Xuzhou, China; ^5^ Department of Endocrinology, Affiliated Hospital of Xuzhou Medical University, Xuzhou, China; ^6^ General Practice, Affiliated Hospital of Xuzhou Medical University, Xuzhou, China; ^7^ Department of Plastic Surgery, Affiliated Hospital of Xuzhou Medical University, Xuzhou, China; ^8^ Follow-Up Office, Affiliated Hospital of Xuzhou Medical University, Xuzhou, China; ^9^ Department of Gerontology, Affiliated Hospital of Xuzhou Medical University, Xuzhou, China; ^10^ Emergency Medicine Department, Suining People’s Hospital, Xuzhou, China

**Keywords:** dysphagia, stent, oesophagal cancer, radiotherapy, meta-analysis

## Abstract

**Introduction:**

Esophageal cancer is one of the most aggressive malignancies with limited treatment options, thus resulting in high morbidity and mortality. For patients with advanced esophageal cancer, the median survival is 3–6 months, with the majority requiring intervention for dysphagia.

**Objective:**

To compare the relief of dysphagia in patients with incurable esophageal cancer treated with stenting alone or a combination of stenting and palliative radiotherapy.

**Methods:**

The protocol of this study was pre-registered on PROSPERO (CRD42022337481). We searched PubMed, Wan Fang, Cochrane Library, Embase, and Web of Science databases. The literature search, quality assessment, and data extraction were conducted by two reviewers independently. The primary endpoints included median overall survival and dysphagia scores. Bleeding events, stent migration, and pain events were secondary outcomes. The meta-analysis results (the primary and secondary outcomes) were pooled by means of a random-effect model or a fixed-effects model.

**Results:**

Nine studies with a total of 851 patients were included in this meta-analysis, consisting of 412 patients in the stenting alone group and 439 patients in the palliative radiotherapy after esophageal cancer stenting (ROCS) group. The ROCS group could significantly improve dysphagia scores (SMD: −0.77; 95% CI: −1.02 to −0.51) and median overall survival (SMD: 1.70; 95% CI: 0.67–2.72). Moreover, there were no significant differences between the two groups in bleeding events, pain events, and stent migration.

**Conclusion:**

Patients with dysphagia in advanced esophageal cancer may benefit further from ROCS in median overall survival and dysphagia scores. However, there was no significant advantage in improving bleeding events, pain events, and stent migration. Therefore, it is urgent to find a better therapy to improve adverse events in the future.

**Systematic Review Registration:**

https://www.crd.york.ac.uk/prospero/, identifier CRD42022337481.

## Introduction

The incidence of esophageal cancer has rapidly increased over the past years, and it is currently the fifth most common type of cancer worldwide with a very high mortality rate (1–3). There were more than 604,000 people newly diagnosed with esophageal cancer and approximately 544,000 deaths due to esophageal cancer worldwide in 2020, according to the World Health Organization (WHO). A majority of patients present with an incurable disease and rapid progression. Patients with advanced esophageal cancer have a poor quality of life during their limited survival time because of dysphagia and have a median survival of 3–6 months. In addition, patients with advanced esophageal carcinoma had a poor quality of life during their limited survival time because of dysphagia.

The management of dysphagia owing to esophageal cancer is challenging. Several management options have been used for the palliation of dysphagia. As the search for ideal therapy for esophageal carcinoma continues, we focus on improvements in dysphagia, overall survival, and adverse events. This meta-analysis, therefore, aimed to evaluate the usefulness of palliative radiotherapy after esophageal cancer stenting (ROCS) for the treatment of patients with inoperable esophageal cancer. Then, it allows us to achieve a better knowledge of palliative modality treatment for advanced esophageal carcinoma patients. Several management options have been used for the palliation of dysphagia (4). Although chemical and thermal ablation, self-expanding metal stents (SEMS), and radiotherapy and chemotherapy alone or in combination were included as options to fight against esophageal cancer (2), placement of metal stents has been the current traditional intervention. However, stent placement is not complication-free (1, 2). Several randomized controlled trials (RCTs) have been performed to compare different treatments, but no one has shown significant advantages over the others.

Twenty years ago, attempts were adopted to combine radiotherapy with stent placement in patients with esophageal cancer (3). A few studies have reported superior results for ROCS with regard to both the relief of dysphagia and survival in patients with advanced esophageal cancer (5–8). Meanwhile, the risk of stent-related adverse events increases over time. Therefore, the guidelines published recently by the European Society of Gastrointestinal Endoscopy (ESGE) strongly summon palliative radiotherapy as a valid alternative to stenting in patients with dysphagia and longer life expectancy (3, 4).

Despite this strong recommendation, palliative radiotherapy is not fully utilized, possibly because of the unawareness of its usefulness (5, 9). ROCS is rarely used as a monotherapy for the rapid relief of dysphagia, but its use immediately after stenting has not been rigorously studied (2). At the same time, the choice of therapy remains a challenging issue due to individual patient factors that are of great complexity, such as age, tumor burden, baseline performance score, existence of metastases, and expected survival time (10).

## Methods

This study was finished with the Preferred Reporting Items for Systematic Reviews and Meta-Analyses (PRISMA) (11) and was registered on PROSPERO successfully (CRD42022337481).

### Data sources and search strategy

QL and HM conducted a comprehensive literature search to screen relevant full articles evaluating the efficacy of palliative radiotherapy combined with stent insertion to relieve dysphagia in advanced esophageal carcinoma patients.

We searched five electronic databases (PubMed, Wan Fang database, the Cochrane Library, Embase, Web of Science) from inception to 30 April 2022, with the following medical subject headings (MeSH) and keywords including “dysphagia,” “stent OR Self-expandable Metallic Stent,” “oesophageal cancer OR carcinoma,” “inoperable esophageal carcinoma,” “radiotherapy,” and “brachytherapy.” There were no language or date restrictions in this meta-analysis.

### Inclusion/exclusion criteria

Eligible studies regarding patients with a diagnosis of dysphagia secondary to esophageal cancer treated by palliative radiotherapy combined with stent insertion were considered. ZX and JC independently applied the inclusion and exclusion criteria to the articles.

All the included studies met the following criteria:

1) RCTs or observational studies,

2) there were at least 30 patients in all selected studies,

3) the main interventions: patients with inoperable esophageal cancer treated with esophageal stenting alone or a combination of esophageal stenting and radiotherapy,

4) participants were adult (≥16 years old) patients with incurable esophageal carcinoma, and

5) studies included should report at least one of the predefined outcomes: dysphagia, survival, or complications (bleeding, pain, etc.).

The exclusion criteria included retrospective studies and prospective studies with less than 30 patients and studies published only in abstract form. Review articles, duplicate articles, editorials, and letters were excluded.

### Study selection

Two review authors (XL and JC) independently scrutinized all studies by title and abstracts. A full-text review of all screened studies was then assessed to determine whether the studies fulfilled the inclusion criteria. Any disagreements were sorted out through discussion with all the authors.

### Data extraction and study quality

Two reviewers (HNL and ZX) independently extracted the data from all included studies. Disagreements were resolved by discussion and consensus with the corresponding authors.

The authors used a standardized data extraction form containing the following items: first author, publication year, study characteristics (RCTs, retrospective and prospective studies), country, sample size, stent diameter, radiotherapy regimen, primary outcomes, the publication status, the study design and location, the number of centers involved, and the Score of the Newcastle Ottawa Quality Assessment Scale (NOS).

### Risk-of-bias assessment

**Figure 1:**
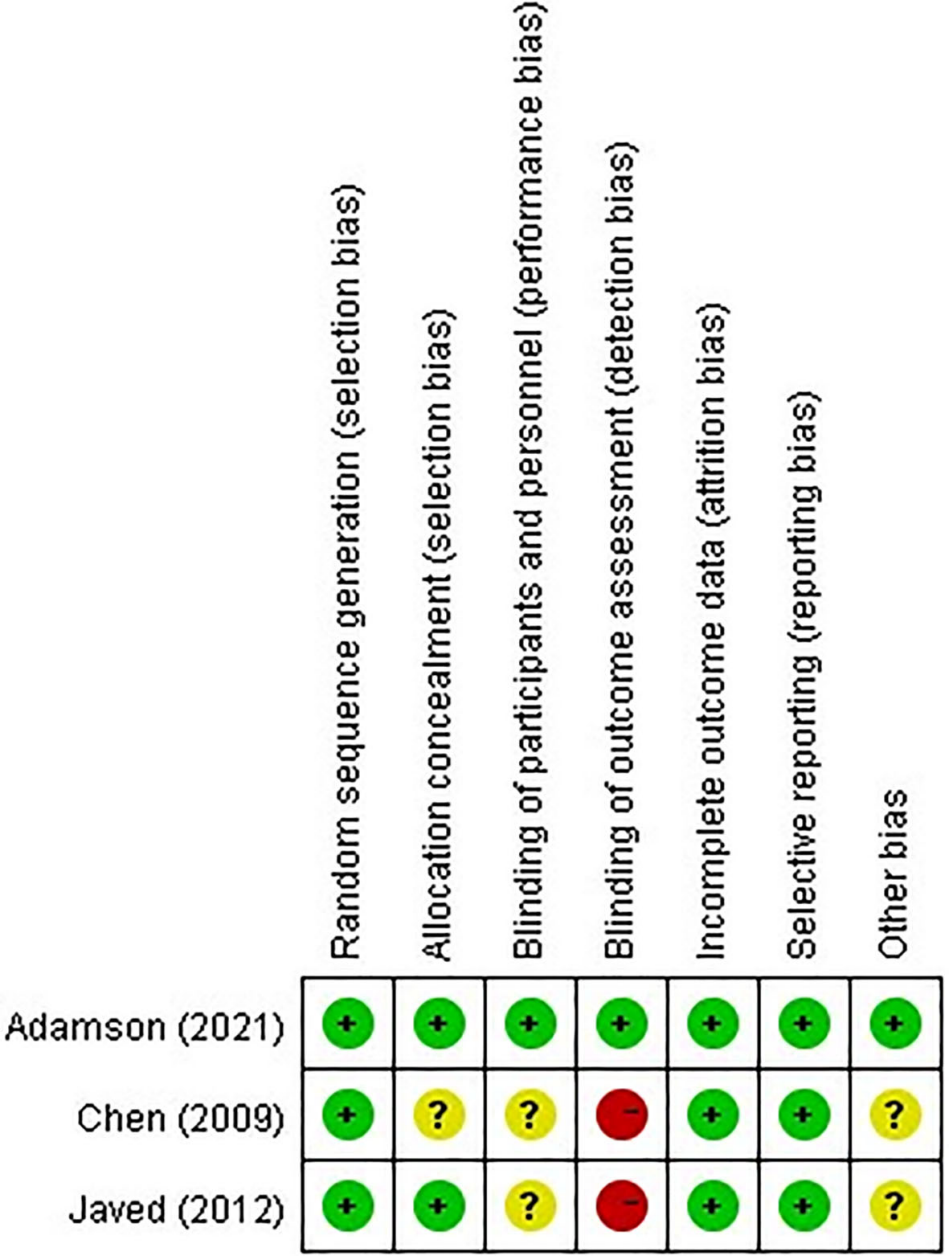
Risk bias assessment in the studies included.

XL and JY assessed the risk of bias of the selected studies independently using the Cochrane risk-of-bias tool and the NOS. To assess the risk of bias within the included randomized trials, the methodological quality of potential studies was evaluated according to the Cochrane risk-of-bias tool (**Figure 1**).

The quality of observational studies was determined according to NOS. The difference in point of view was resolved by consulting the third researcher (XY).

### Statistical analysis

We (SL and HNL) used the Stata Statistical Software (version 12.0; Stata Corp., College Station, TX, USA) and Review Manager (version 5.4; The Nordic Cochrane Center, The Cochrane Collaboration, Copenhagen, Denmark) for all statistical analyses.

The primary and secondary outcomes were pooled by means of a random-effects model or a fixed-effects model (12). We integrated the dichotomous variables as risk ratios (RRs) with 95% confidence intervals (CIs) and continuous variables as standardized mean differences (SMDs) with 95% CI. Statistical heterogeneity was calculated by I2 tests, with I2 >50% being indicative of significant heterogeneity. The potential publication bias was assessed by visual inspection of Begg’s funnel plot. Begg’s rank correlation test and Egger’s linear regression test were also evaluated at the p <0.10 level of significance (13, 14). All tests were two-sided and a p-value less than 0.05 was considered statistically significant.

## Results

### Search results and trial characteristics

**Figure 2:**
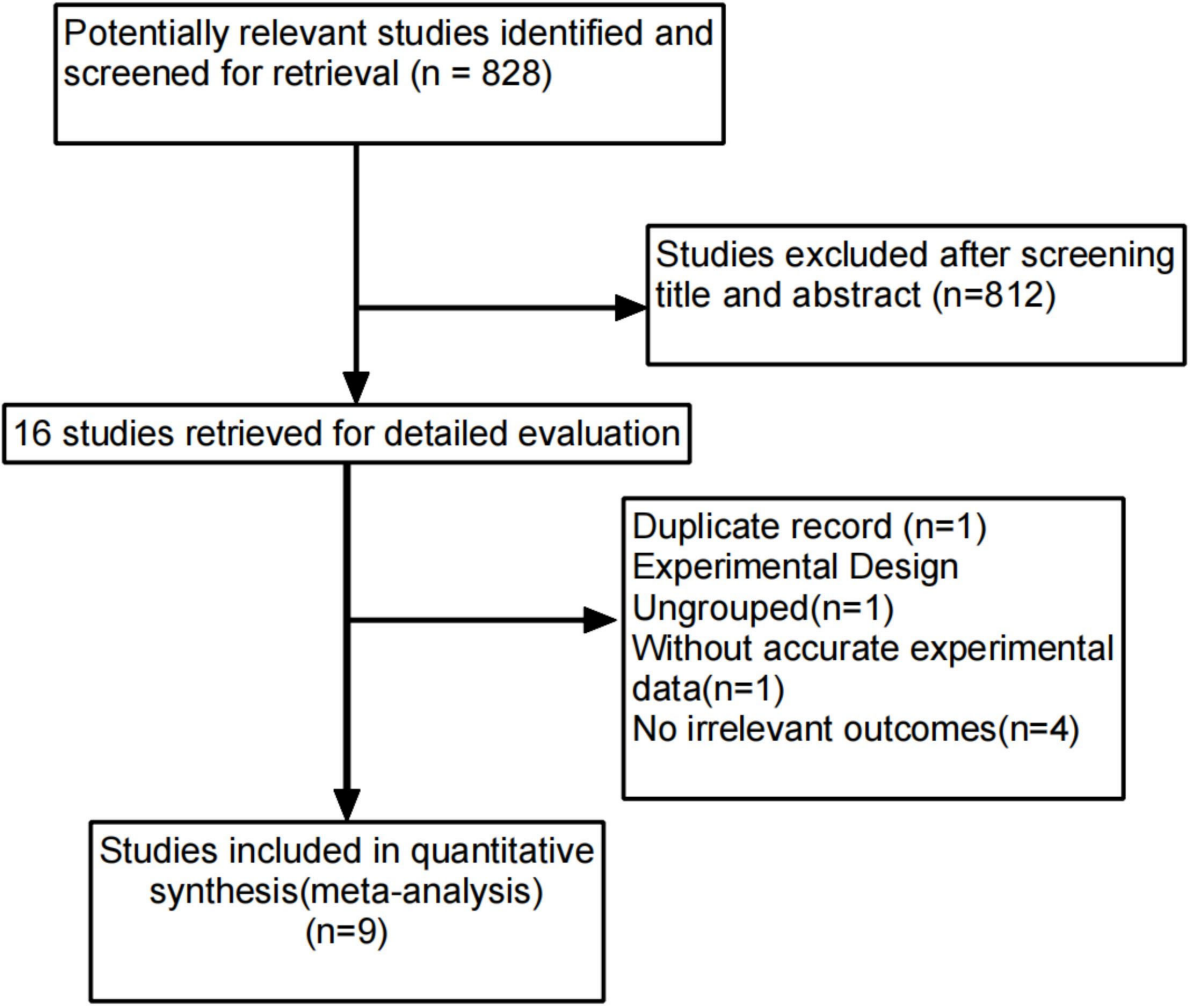
Process for identifying studies eligible for the meta-analysis.

A total of 828 studies were identified through the systematic search. Eight hundred and twelve studies were excluded after screening the title and abstract, and 16 studies remained available. Seven articles were excluded for the following reasons: one article was related to duplicate data, one article did not include a control group, four articles did not provide relevant outcomes, and one article did not provide accurate experimental data. Nine studies with a total of 851 patients were included in the meta-analysis (**Figure 2**). The study characteristics are listed in **Table 1**. Of all nine studies, three studies were from Western countries (15, 17, 18), and six studies were from multiple areas (6, 16, 19–22). This meta-analysis included three RCTs (15, 16, 22) and six observational studies (6, 17–21). The sample size ranged from 34 to 220 patients. Median survival was the primary endpoint for most studies. A total of 851 patients were included in the meta-analysis, of which 439 patients were in the adjuvant external beam radiotherapy group and 412 patients were in the usual care alone group.

**Table 1:** Summary of the identified nine studies.

Study	Year	Country	Research type	Sample size	Stent diameter (mm)	Radiotherapy regimen	Primary outcome	Scores of NOS	Scores of jaded
Adamson (15)	2021	UK	Randomized controlled study	220	Not given	20 Gy in five fractions or 30 Gy in 10 fractions	Median survival time	–	5
Javed (16)	2012	India	Randomized controlled study	84	18	30 Gy in 10 fractions	Median survival time	–	4
Eldeeb (17)	2012	UK	Prospective study	91	Not given	20 Gy in five fractions or 30 Gy in 10 fractions	Median survival time	8	–
Rueth (18)	2012	USA	Retrospective study	37	Not given	Not given	Median survival time	8	–
Song (19)	2002	China	Prospective study	108	16	Not given	Median survival time	7	–
Zhong (6)	2003	China	Prospective study	34	18	1.8 to 2.0 Gy for each session, 45 to 55 Gy totally	Dysphagia scores	6	–
Xie (20)	2002	China	Prospective study	47	Not given	1.8 Gy for each session, 45 to 55 Gy totally	Dysphagia scores	6	–
Ao (21)	2012	China	Retrospective study	150	Not given	2 Gy each time, 5 times a week	Dysphagia scores	7	–
Chen (22)	2009	China	Randomized controlled study	80	Not given	1.8 Gy for each session, 45 to 55 Gy totally	Median survival time	–	4

### Median overall survival

**Figure 3:**
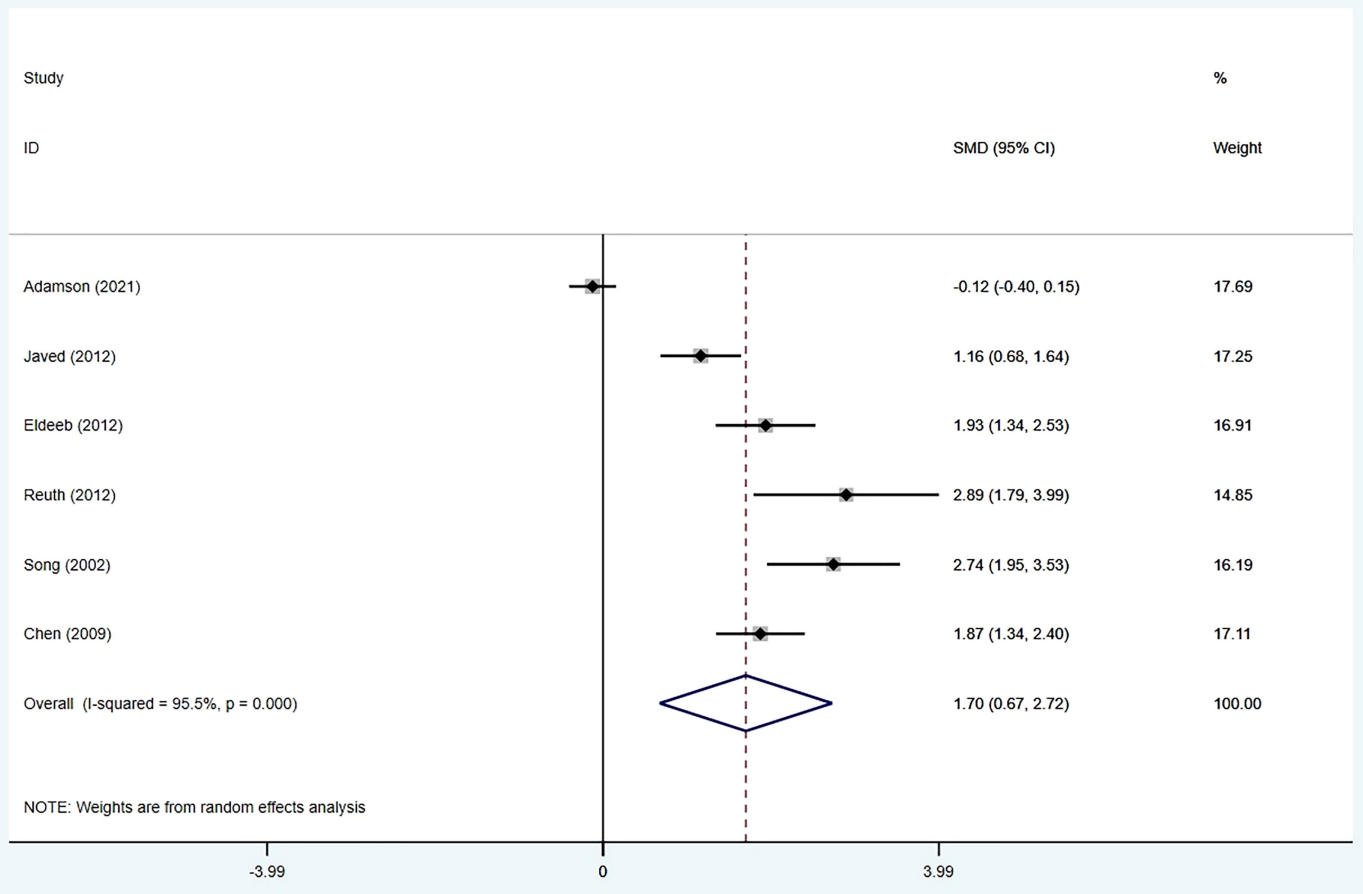
Forest plot for the results of median overall survival.

Six studies (15–19, 22) provided data on the median overall survival. We found that ROCS had a significantly prolonged median overall survival compared with stenting alone (SMD: 1.70; 95% CI: 0.67–2.72). Significant heterogeneity was found among the six studies (I2 = 95.5%; p < 0.001) (**Figure 3**).

### Dysphagia scores

**Figure 4:**
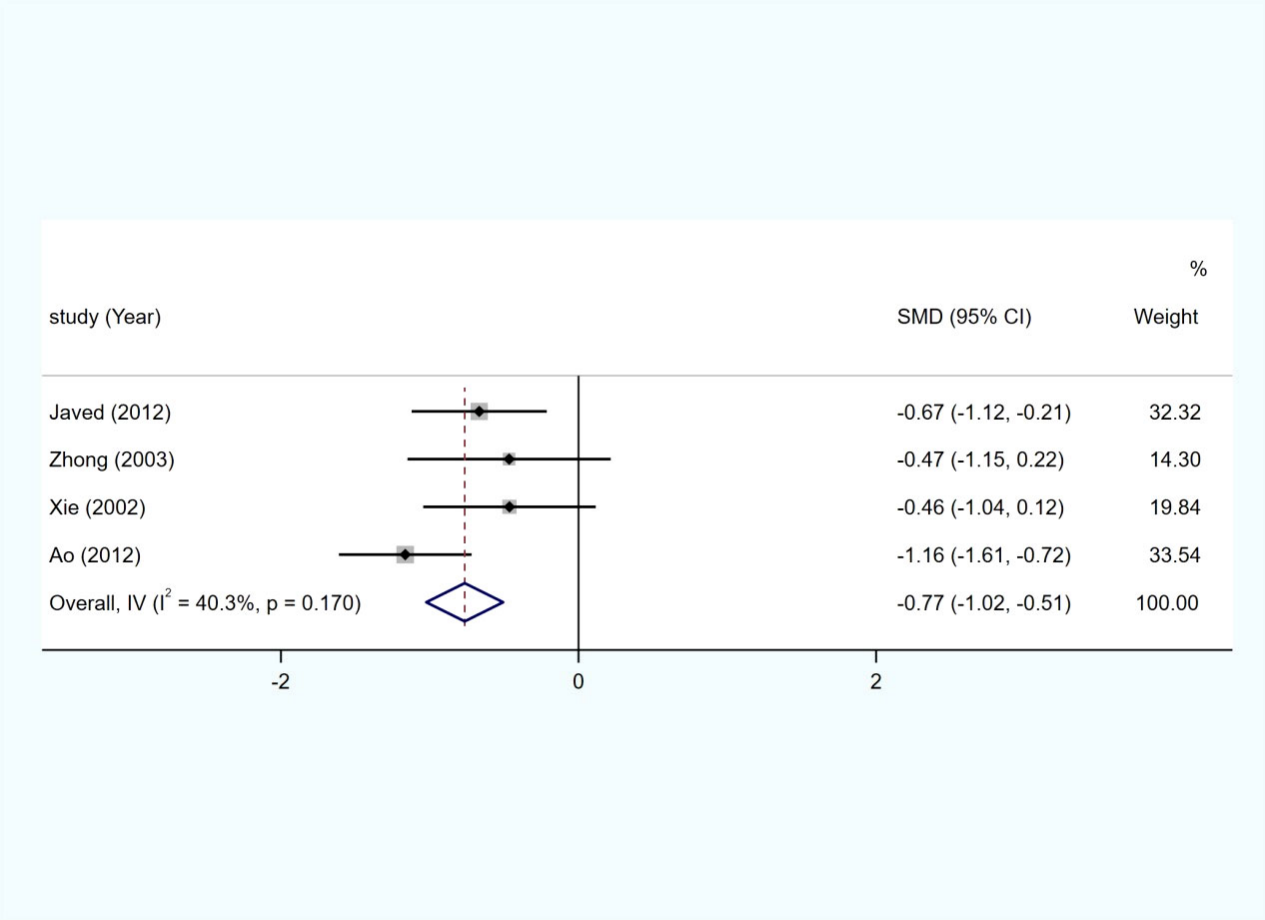
Forest plot for the results of dysphagia scores.

Four studies (6, 16, 20, 21) provided data on the dysphagia scores. The data for the dysphagia scores were available in four articles. The pooled results indicated that patients receiving ROCS showed significantly better dysphagia scores than patients receiving stenting alone (SMD: −0.77; 95% CI: −1.02 to −0.51) with no significant heterogeneity (I2 = 40.3%; p = 0.170) (**Figure 4**).

### Bleeding events

**Figure 5:**
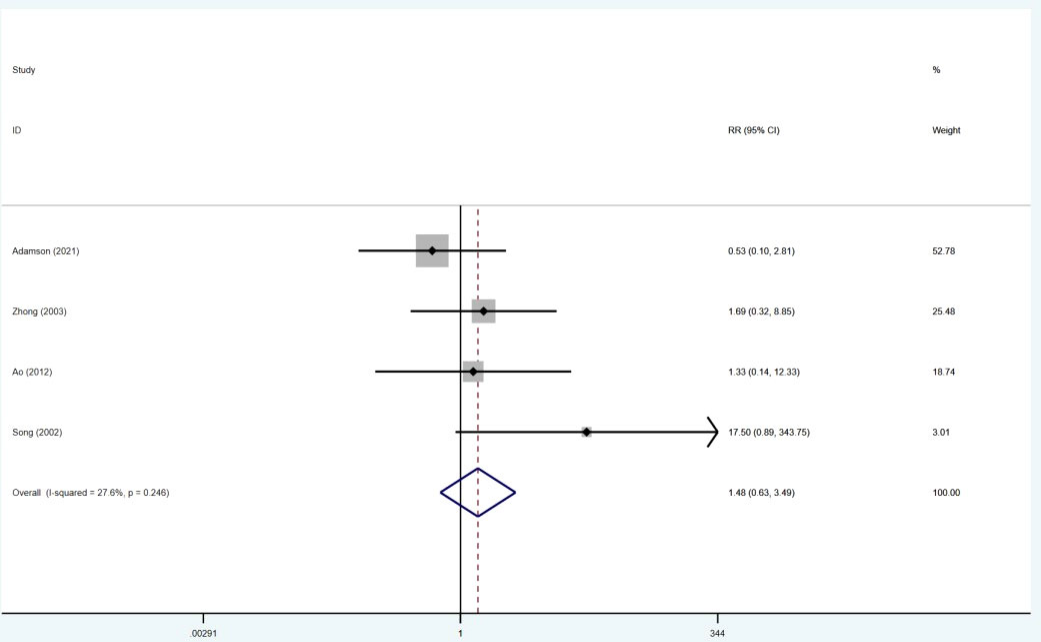
Forest plot for the results of bleeding events.

The data for bleeding events were extracted from four studies (6, 15, 19, 21). There was no significant difference in bleeding between the two groups (RR = 1.48; 95% CI: 0.63–3.49). No significant heterogeneity studies were observed between these studies (I2 = 27.6%; p = 0.246) (**Figure 5**).

### Pain events

**Figure 6:**
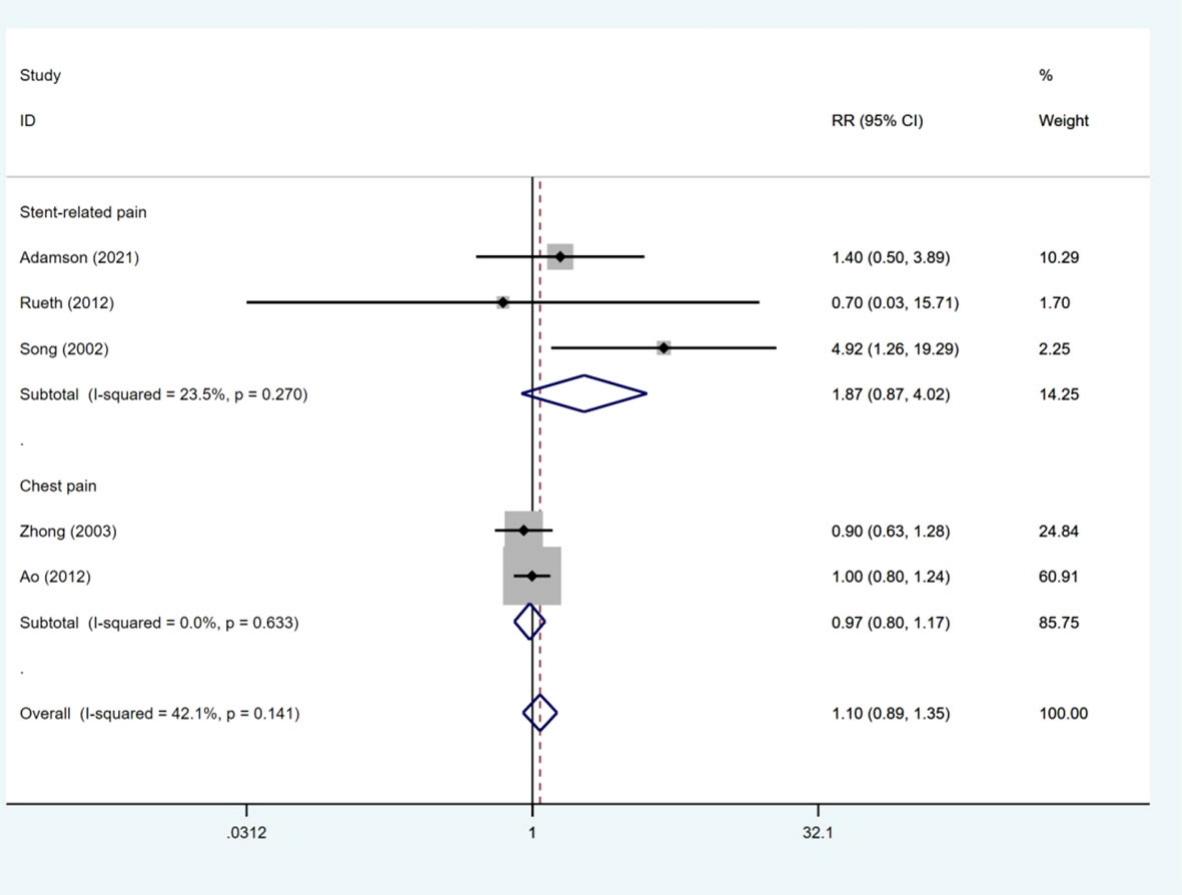
Forest plot for the results of pain events.

Five studies (6, 15, 18, 19, 21) presented data on pain events. The number of pain events was much the same between the ROCS group and the stenting alone group (RR: 1.10; 95% CI: 0.89–1.35). No heterogeneity was observed between studies (I2 = 42.1%, p = 0.141). Subgroup analysis was used to evaluate the RR of pain events based on different types of pain events. As shown in **Figure 4**, the RRs of stent-related pain and chest pain were 1.87 (95% CI: 0.87–4.02) and 0.97 (95% CI: 0.80–1.17), respectively (**Figure 6**).

### Stent migration

**Figure 7:**
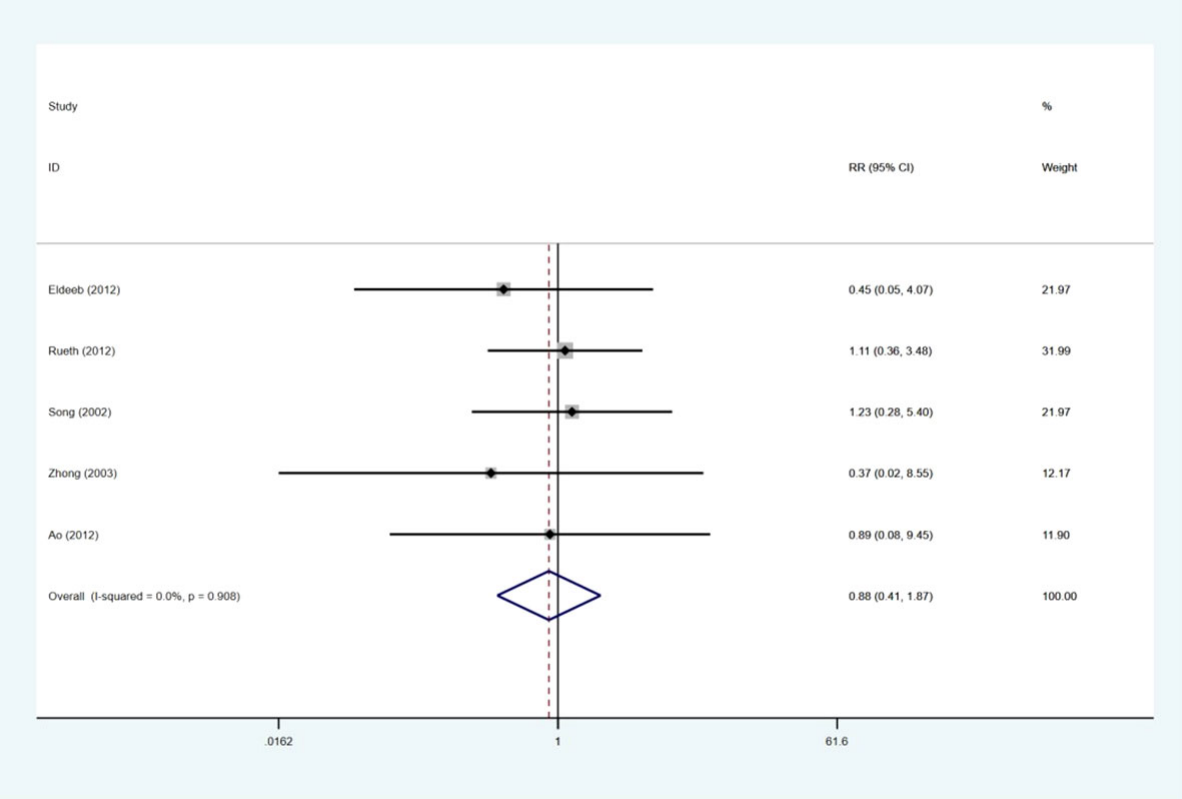
Forest plot for the results of stent migration.

Five of the study reports (6, 17–19, 21) revealed the data on stent migration. We discovered that there were comparable pooled stent migration events between the two groups (RR: 0.80; 95% CI: 0.41–1.87), and no significant heterogeneity was apparent among the five studies (I2 = 0.0%; p = 0.908) (**Figure 7**).

### Publication bias

**Figure 8:**
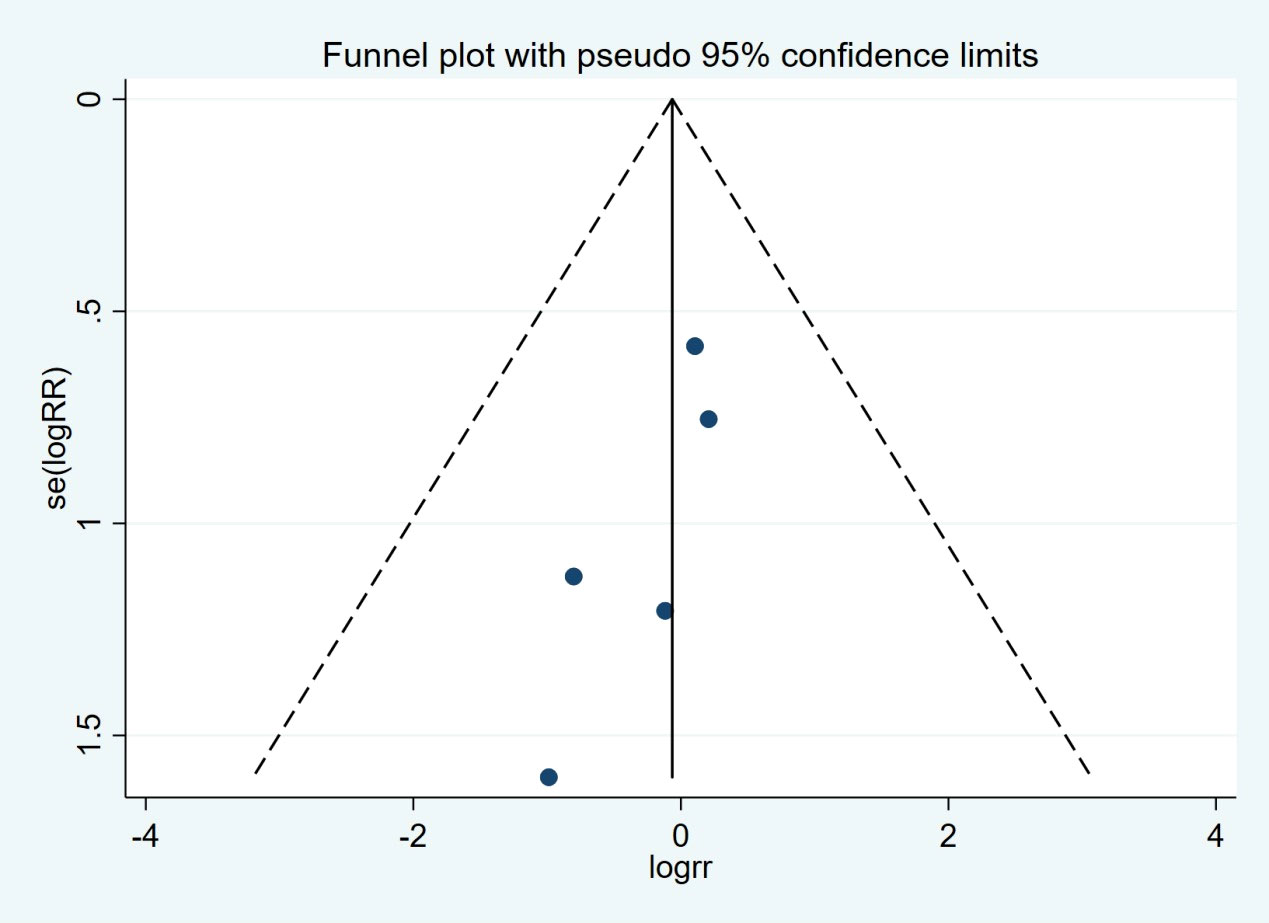
Funnel plots for stent migration.

There was no evidence of publication bias by inspection of the funnel plot and statistical tests (Begg’s test, p = 0.462; Egger’s test, p = 0.118) (**Figure 8**).

## Discussion

Of the nine trials included in this study, the clinical efficacies were primarily evaluated by comparing the median overall survival and dysphagia scores. Our results showed that ROCS can significantly improve median survival and dysphagia scores compared with the control group. However, ROCS group did not show significant improvement of related complications, such as bleeding events, stent migration, and pain events. In the majority of cases, the diagnosis of esophageal carcinoma occurs at an advanced stage (1). Despite the prevalence and impact of dysphagia in esophageal cancer, no systematic review has previously been attempted to summarize the evidence for palliative radiotherapy combined with stent insertion to relieve dysphagia in advanced esophageal carcinoma patients. Dua (2) found that esophageal stents were a very effective treatment for relieving dysphagia, with an effective rate of 96% to 100%. In this meta-analysis, we appraised the reported clinical efficacies of palliative radiotherapy after ROCS for treating patients with dysphagia in advanced esophageal carcinoma.

The first-line treatment option is generally represented by stent placement because of the simplicity of this procedure and the prompt resolution of dysphagia after stent deployment, which is achieved in almost all cases within 2 days (1). Stent placement is not complication-free, and the overall incidence of severe adverse events seems to be comparable to that of palliative ROCS (3). The efficacy of stenting tends to decrease over time; therefore, the most recent international guidelines recommend brachytherapy for patients with longer life expectancy (i.e., >4 months) (5, 23). The role of ROCS remains controversial. Indeed, in the study by Sur et al. published in 2004, the authors did not report any significant difference concerning the dysphagia-free survival (DFS) at 6 and 12 months after treatment between the groups receiving either stent alone or ROCS (10), while the study by Rosenblatt et al., published in 2010, showed a significant improvement of the DFS in the group of patients treated by ROCS (24). Therefore, the role of stent alone or ROCS in dysphagia in advanced esophageal carcinoma patients is still not clear, and further studies should investigate this issue. It is worth noting that substantial heterogeneity in the initial palliative approach of patients with inoperable esophageal cancer has been described (14, 25). The paucity of therapeutic guidance is possible to bring about this diversity in the initial treatment; indeed, clinical decision-making should be based not just on patient- and disease-related factors, but it should also be significantly influenced by the hospital of diagnosis (25). Therefore, governments and hospitals should strongly encourage evidence-based treatments and logistical issues contextually resolved to provide the optimal palliative management strategy.

The strengths of this study involved broad inclusion criteria and relevant exclusion criteria to ensure that all relevant studies were included in the review. Our study not only included relevant research on a global scale but also evaluated the relevant projects in strict accordance with the screening criteria corresponding to the topic. Nonetheless, everything has two sides. First, our meta-analysis presented with a considerable number of limitations, involving the heterogeneity of results, due to limited availability of information since only 9 studies were reviewed. Second, in order to pursue the universality of relevant research, some related studies included were conducted long ago, and the quality of evidence for long interval studies comparing stent combination versus stenting alone was also very low, which may increase the heterogeneity of our results. Third, due to the complexity of the work and the diversity of the included studies, we did not conduct further analysis according to subgroups, and the patients included in the study had different follow-up years and distinct countries and nationalities, which further increased the heterogeneity of the results. Fourth, different treatment methods may also increase the heterogeneity of the research results. For example, in these projects included in our study, the doses and cycles of radiotherapy were not exactly the same. Fifth, different types of stent treatment may also have an impact on the results of the study, such as the material of the stents, the shape of the stents, the diameter of the stents, and so on. Sixth, there are wide confidence intervals for the pooled analyses of adverse events, which highlighted the lack of event data to draw meaningful conclusions. Above all, the types and sizes of esophageal tumors may affect the median survival time and complications after treatment, and subgroup analysis was not conducted in our study due to their diversity.

## Conclusion

In conclusion, our meta-analysis demonstrated that patients with advanced esophageal cancer might benefit further from ROCS in median overall survival and dysphagia scores. However, there was no significant advantage in improving bleeding events, stent migration, and pain events. Future research should focus on combined therapy, which can alleviate adverse events. It is of great desirability that more RCTs are conducted to confirm the effects of the two groups of treatment.

## Data availability statement

The datasets presented in this study can be found in online repositories. The names of the repository/repositories and accession number(s) can be found in the article/supplementary material.

## Author contributions

ZX and HL: writing of this paper and collection of related research literature. JY, JC, and XL: extraction and proofreading of relevant data. QL and HM: supervision and improvement of article language quality. SL: guidance and supervision of statistical methods. PJ and XY: design of this study as well as funding support. All authors contributed to the article and approved the submitted version.

## Funding

This research was supported by the Excellent Teaching Team of “Qinglan Project” in Jiangsu, Xuzhou National Clinical Key Specialty Cultivation Project (2018ZK004); Jiangsu Provincial Commission of Health and Family Planning (LGY2019085); The Excellent Young and Middle-Age Talents Project of the Affiliated Hospital of Xuzhou Medical University (2019128009); and the National Keypoint Research and Invention Program (2020YFC1512704).

## Acknowledgments

We thank SL (a professor of statistics and a senior statistician) and HLL for their guidance and supervision on the statistical methods of this study. Professor ZH provided professional advice and guidance on the knowledge related to esophageal cancer. We are grateful to professor XY and PJ for their funding support and technical guidance.

## Conflict of interest

The authors declare that the research was conducted in the absence of any commercial or financial relationships that could be construed as a potential conflict of interest.

## Publisher’s note

All claims expressed in this article are solely those of the authors and do not necessarily represent those of their affiliated organizations, or those of the publisher, the editors and the reviewers. Any product that may be evaluated in this article, or claim that may be made by its manufacturer, is not guaranteed or endorsed by the publisher.
